# The Hoover's Sign of Pulmonary Disease: Molecular Basis and Clinical Relevance

**DOI:** 10.1186/1476-7961-6-8

**Published:** 2008-09-05

**Authors:** Chambless R Johnston, Narayanaswamy Krishnaswamy, Guha Krishnaswamy

**Affiliations:** 1 Department of Internal Medicine, Quillen College of Medicine and James. H. Quillen VA Medical Center, Johnson City, TN 37614-0622, USA; 2 Department of Medicine, Division of Allergy and Clinical Immunology, James. H. Quillen VA Medical Center, Mountain Home, TN 37684, USA

## Abstract

In the 1920's, Hoover described a sign that could be considered a marker of severe airway obstruction. While readily recognizable at the bedside, it may easily be missed on a cursory physical examination. Hoover's sign refers to the inspiratory retraction of the lower intercostal spaces that occurs with obstructive airway disease. It results from alteration in dynamics of diaphragmatic contraction due to hyperinflation, resulting in traction on the rib margins by the flattened diaphragm. The sign is reported to have a sensitivity of 58% and specificity of 86% for detection of airway obstruction. Seen in up to 70% of patients with severe obstruction, this sign is associated with a patient's body mass index, severity of dyspnea and frequency of exacerbations. Hence the presence of the Hoover's sign may provide valuable prognostic information in patients with airway obstruction, and can serve to complement other clinical or functional tests. We present a clinical and molecular review of the Hoover's sign and explain how it could be utilized in the bedside and emergent management of airway disease.

## Introduction

In the 1920's, Hoover described a sign that could be considered a marker of severe airway obstruction. While readily recognizable at the bedside, it may also as easily be missed on physical examination. Hoover's sign refers to the inspiratory retraction of the lower intercostal spaces. It results from alteration in dynamics of diaphragmatic contraction due to hyperinflation, resulting in traction on the rib margins by the flattened diaphragm. The sign is reported to have a sensitivity of 58% and specificity of 86% for detection of airway obstruction. Seen in up to 70% of patients with severe obstruction, this sign is often associated with body mass index, degree of dyspnea and frequency of exacerbations. Often overlooked, Hoover's sign may provide valuable prognostic information. When present, the sign can be used, along with arterial blood gasses, pulmonary function and other measures summarized in Table 1, as a marker for severity of airway obstruction, as seen in emphysema, chronic obstructive pulmonary disease (COPD) or asthma.

**Table 1:** Suggested Indices Of Severity Of Airway Obstruction

**Physical findings**	Pursed lip breathing
	Intercostal retraction (Hoover Sign)
	Accessory muscle use
	Cyanosis
	**Hoover's sign?**
	
**Laboratory Parameters**	Pulmonary function (FEV_1 _and FEV_1_/FVC)
	Peak Expiratory Flow Rate
	Hypoxemia

Better clinical and bedside prognosticators of airway obstruction would be helpful as asthma and COPD are becoming increasingly prevalent in the population [1]. COPD is the fourth leading cause of death in the United States behind coronary artery disease, malignancy, and cerebrovascular disease. In 2000, an estimated 10 million US adults reported physician-diagnosed COPD. Data from the Third National Health and Nutrition Examination Survey (NHANES III), however, estimate that among 11 million US adults with evidence of low lung function, < 40% reported a diagnosis of COPD or asthma, suggesting that COPD is under-diagnosed. Acute exacerbations of COPD can result in ventilator failure, and patients with severe COPD or asthma are more prone to developing this complication. A clinical, quickly identified manifestation of respiratory failure is the Hoover's sign, which does not require expensive tests or waiting for radiological or biochemical results (such as arterial blood gases). Moreover, when patients presents with an acute exacerbation of airway disease in the emergency room or in a physician's office, they are less likely to tolerate laborious radiological examinations (such as computed tomograms) and pulmonary function tests (which require intense patient participation). It is in this situation that a positive Hoover's sign, in association with other clinical parameters, blood gases or peak expiratory flow tests is likely to assist in patient triage and management in emergency settings. We present a review of the clinical and molecular/structural basis of the Hoover's sign and explain how it could be utilized in the bedside and emergent management of severe airway disease.

### Clinical presentation of Hoover's sign

#### Case Report

**Figure 1:**
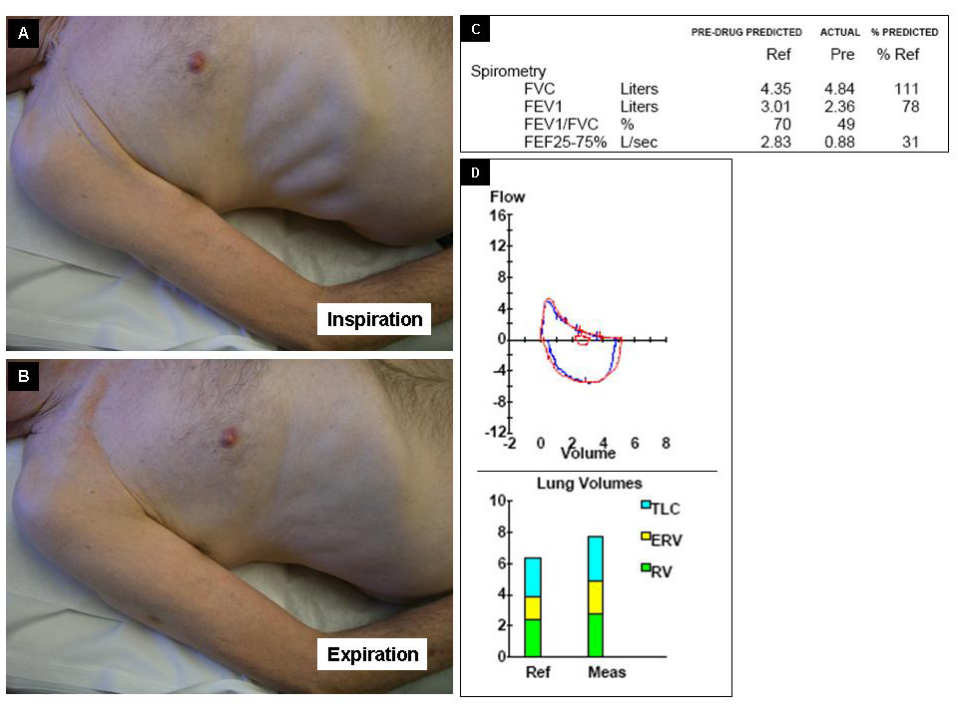
**Hoover's sign refers to the paradoxical inspiratory retraction of the rib cage and lower intercostal interspaces **(**Figure 1 Panels A and B**). This patient had evidence of moderate airway obstruction and elevated residual volumes (**Figure 1 Panels C and D**).

**Figure 2:**
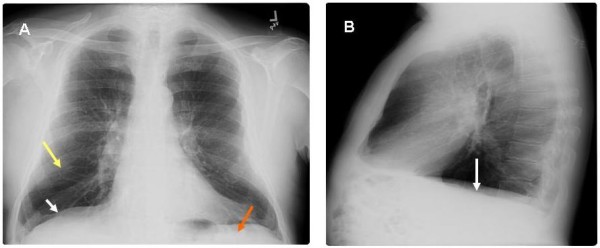
**With A showing PA and B showing lateral views of the chest roentgenograms of the same patient. **The arrow marks refer to the flattening of the diaphragm (white arrows), emphysematous changes (yellow arrow) and the decreased zone of apposition (red arrow).

Figure 1 demonstrates the chest wall findings in a 65 year old male long-term smoker who had frequent hospitalization for wheezing in spite of oral steroids. The patient's medications included prednisone (20 mg/day), formoterol and lisinopril. Examination revealed a thin, dyspneic Caucasian male. Pursed lip breathing, bilateral expiratory wheezing and Hoover's sign were present. Hoover's sign refers to the paradoxical inspiratory retraction of the rib cage and lower intercostal interspaces (Figure 1 Panels A and B). This patient had evidence of moderate airway obstruction and elevated residual volumes (Figure 1 Panels C and D). There was poor reversibility with bronchodilators. The patient had a low alpha-1 antitrypsin level of 83 mg/dl (N = 90–200 mg/dl) and he was classified as a MZ phenotype. Figure 2 demonstrates the chest roentgenogram of the patient, with panel A being the postero-anterior and B lateral views of the chest roentgenograms of the same patient. The arrow marks refer to the flattening of the diaphragm (white arrows), emphysematous changes (yellow arrow) and the decreased zone of apposition (red arrow). The significance of this is discussed under mechanisms below.

### What is Hoover's sign?

Originally described in 1920 by Hoover, this eponymous sign refers to the paradoxical inspiratory indrawing of the lateral rib margin which has been attributed to direct traction on the lateral rib margins by the flattened diaphragm [2,3]. Normally, the costal margin moves very little during regular breathing, but, if it does, it moves outward and upward. In patients with obstructive airway disease there is a higher tendency for it to move paradoxically [4]. In these patients, paradoxical movements of the sternum as well as of the abdominal wall may be seen [5]. Garcia-Pachon et al., found Hoover's sign expression in 62 out of 82 patients with COPD (sensitivity of 76%), 3 out of 23 patients with asthma (13%) and in 3 out of 101 (3%) of patients with congestive heart failure [6]. In a larger study of 157 patients, the same investigators demonstrated presence of Hoover's sign in 71 patients (45% of study population), and in 36%, 43% and 76% respectively of patients with mild, moderate or severe COPD [3]. Garcia-Pachon also showed that patients with COPD and Hoover's sign tended to have a higher dyspnea index/score, have higher hospitalizations or emergency room visits than patients without the sign [7]. It appears that Hoover's sign may provide excellent prognostication of severe COPD. In a multivariate analysis, severity of dyspnea, the patient's body mass index, numbers of exacerbations historically and numbers of prescribed drugs were independently associated with the sign [3].

The Hoover's Sign of Hysterical Paralysis, not to be confused with the sign being discussed, can be found in the neurological literature that describes a sign to separate organic from non-organic paresis of the leg. Involuntary extension of the paralyzed leg occurs when flexing the contralateral leg against resistance. The patient lies supine, the examiner's hand is placed under the non-paralyzed heel, and the patient is asked to elevate the paralyzed leg. In organic paresis the examiner feels a downward pressure under the non-paralyzed heel; in malingering no pressure is felt. This sign is not within the purview of the current review.

### Presumed molecular mechanisms behind Hoover's sign

**Figure 3:**
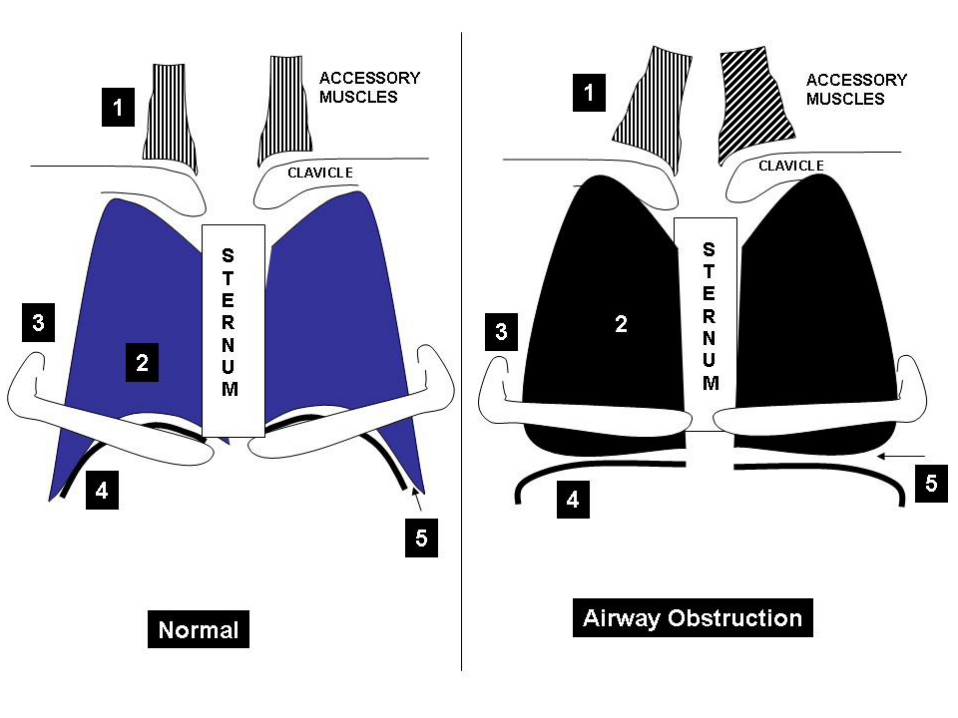
**Demonstrates the mechanism behind Hoover's sign. **The numbers on the figure refer to the following: 1 = accessory muscles, 2 = hyper-expansion of the lungs, 3 = alteration of rib orientation to horizontal 4 = flattened diaphragm and 5 = decreased zone of apposition (adapted from Mason: Murray and Nadel's Textbook of Respiratory Medicine, 4^th ^Edition).

Studies by Gilmartin and Gibson suggest that transdiaphragmatic pressures play a major role in the pathogenesis of Hoover's sign [8]. Figure 3 demonstrates the possible mechanism behind Hoover's sign. With emphysema secondary to airway obstruction, flattening of the diaphragm occurs (as shown also in Figure 2). This leads to increased radius of curvature, which in turn increases muscle tension. Secondary to the horizontal orientation of the diaphragm and the associated loss of the zone of apposition between the visceral and parietal pleurae (Figure 3 right panel), the force vector on the lower aspects of the ribs become inward rather than cephalad. This culminates in the lower rib cage motion directed inward on inspiration instead of outward, the paradoxical movement referred to as Hoover's sign. In an exacerbation, the presence of mucus and bronchoconstriction further increases airway resistance, work of breathing and lung inflation. This leads to more diaphragmatic flattening and exacerbation of the mechanisms mentioned above. It would be interesting to study molecular changes in the musculature such as expression of certain muscle genes and ultrastructural alterations in muscle but these have not been done.

### Clinical significance of Hoover's sign

Hoover's sign is a frequent finding in COPD, and the frequency increases with severity. The sign can also be present in patients with congestive heart failure, asthma, severe pneumonia (especially in children), bronchiolitis, as well as seen unilaterally in diaphragmatic paralysis, pleural effusion and pneumothorax.

The Hoover's sign is reported to have a sensitivity of 58% and specificity of 86% for detection of airway obstruction in a study by experienced respiratory medicine specialists among a group of first year residents in family medicine [9]. The study compared the accuracy of Hoover's sign detecting obstructive airway disease compared with traditional signs such as wheezing, rhonchi and/or reduced breath sounds. Observer agreement in the study (kappa statistic) was 0.74 for Hoover sign and was lower for the rest of the signs stated above [9]. The Hoover's sign had a positive likelihood ratio of 4.16, which was higher than that of the other signs. Obstructive airway disease in the study was defined as an FEV1/FVC ratio of < 0.70. There have been no studies conducted on the sensitivity and specificity of Hoover's sign in asthma. There is no data available either on the cost savings that may be induced by using Hoover's sign as opposed to use of chest roentgenography, pulmonary function tests or arterial blood gases, for example. The duration of persistence of Hoover's sign, its appearance or disappearance in relationship to exacerbations and remissions and the influence of aggressive therapy on extent of retraction are hitherto unknown. Further studies would certainly improve insights into the pathogenesis of airway obstruction but probably would be unlikely to be done in this day and age of high technology and digital imaging.

## Conclusion

Hoover's sign refers to the inspiratory retraction of the lower intercostal spaces. It results from alteration in dynamics of diaphragmatic contraction due to hyperinflation, resulting in traction on the rib margins by the flattened diaphragm. Seen in up to 70% of patients with severe obstruction, this sign is associated with body mass index, dyspnea and frequency of exacerbations. This sign can be an excellent marker for severe airway obstruction.

## Competing interests

The authors declare that they have no competing interests.

## Consent

Written informed consent was obtained from the patient for publication of this case report and accompanying images. A copy of the written consent is available for review by the Editor-in-Chief of this journal.

## Authors' contributions

CRJ carried out the research into Hoover's sign, and in structuring and outline of the manuscript; NK assisted with the case report and editing process; GK conceived the study, helped in the editing process, created the graphics and elaborated the case report.
